# Demonstration of Functional Heterogeneity of T lymphocytes and Identification of Their Two Major Subsets

**DOI:** 10.3389/fimmu.2014.00609

**Published:** 2014-12-01

**Authors:** Paweł Kisielow

**Affiliations:** ^1^Ludwik Hirszfeld Institute of Immunology and Experimental Therapy, Wrocław, Poland

**Keywords:** T lymphocytes, CD4^+^8^−^ and CD4^−^8^+^ subsets, CD4^+^8^+^ thymocytes, positive selection, negative selection

Cells responsible for the specificity of the immune response and thymus function remained unknown until mid 1960s when lymphocytes were identified as mediators of cellular and humoral immunity (1–4). Soon afterward *in vivo* reconstitution experiments showed that cellular and humoral immunity are mediated by two different types of lymphocytes: thymus-derived T cells and thymus-independent, bone marrow derived antibody producing B cells, respectively (5). This finding created a need for a simple and rapid method to distinguish these two classes of lymphocytes, and the immune system itself provided the best tool for this task. In 1969, Martin Raff (6) showed that antibodies to Thy1 antigen [cell surface molecule expressed chiefly in the thymus and brain (7)] react with all thymus-dependent but not with thymus-independent lymphocytes. This allowed the distinguishing and separation of functionally different but morphologically identical lymphocytes allowing study of their specific activities. Meanwhile Edward Boyse, Lloyd Old, and colleagues identified two other cell surface antigens expressed mainly in the mouse thymus, Ly1 and Ly2 (8), which were thought to characterize all T cells, like Thy1.

By that time T cells were known to have several functions and an important question emerged: do different functions reflect their functional heterogeneity or are manifestations of homogenous population of multifunctional cells?

Late in 1972, I obtained a postdoctoral WHO fellowship. Being interested in lymphocyte biology and cell surface immunogenetics I contacted Dr. Lloyd Old, who thanks to the recommendation of my PhD supervisor, Professor Czesław Radzikowski invited me to his laboratory at the Sloan-Kettering Institute in New York. During our first conversation Dr. Old suggested that I join Hiroshi Shiku, another postdoc, to study the mechanism of target cell killing by T cells. Dr. Old proposed to use a panel of antisera known to react with different surface antigens on thymocytes, to find if they could block cytotoxicity and in this way learn about possible involvement in this process of thus identified molecules. Dr. Shiku had just applied to the mouse system a new *in vitro* method, developed to evaluate cytotoxicity of lymphocytes by measuring the remaining radioactivity of adherent target cells labeled with tritiated proline (9). The specific antisera were provided by Dr. Boyse, among them anti Ly1 and anti-Ly2. The only information we had at that time about their reactivity with lymphocytes in different lymphoid organs was derived from *in vitro* absorption assays, which indicated that thymus had higher absorption capacity than lymph nodes and lymph nodes higher than spleen (8) correlating with the number of Thy1 positive cells in these tissues. My task was then to test the antisera against the effector cell population to determine their optimal titers using a complement dependent, trypan blue exclusion cytotoxicity assay.

Our repeated attempts to block cytotoxicity by pre-incubation of allo-reactive effector cells from immunized mice were unsuccessful^1^ but because I noticed that the proportions of lymphocytes lysed by Thy1, Ly1, and Ly2 antisera in lymph nodes, spleen, and thymus were slightly different, we decided to test the effect of elimination of the cells sensitive to these antisera on the ability of surviving cells to kill target cells. The results showed that elimination of cells with Ly2 antisera (which lysed less lymphocytes than Ly1 antisera) inhibited cytotoxicity significantly stronger than elimination of lymphocytes with Ly1 antisera, suggesting that cytotoxic T cells can be distinguished from other T cells by their Ly1^low(−)^Ly2^high(+)^ surface phenotype. Some doubts, however, still remained because the methods used were not sufficiently precise to be sure that the relatively small differences we observed were real. Moreover, since we only tested cytotoxicity (10), the proof of functional heterogeneity of T cells was missing. In order to find whether Ly1^−^2^+^ and Ly1^+^2^−^ lymphocytes may have different functions, I approached John Hirst (who in the Herbert Oettgen laboratory studied the helper activity using the Mishell-Dutton cell culture method) and proposed to test the effect of elimination of lymphocytes with Ly antisera on this regulatory function. Already, the first experiment produced clear-cut results: we observed practically no inhibition by treatment with Ly2 antisera and almost complete inhibition with Ly1 antisera, indicating that helper and killer T cells have different Ly phenotypes: Ly1^+^2^−^ and Ly1^−^2^+^, respectively. Importantly, I found that lymphocytes with different sensitivity to Thy1, Ly1, and Ly2 antisera are also present in the thymus of non-immunized normal mice, strongly suggesting that lymphocytes with different Ly phenotypes are generated independently of antigenic stimulation. These findings (11) represented the first unequivocal demonstration of functional heterogeneity of T cells and identified – as subsequent studies showed – their two major subsets. Moreover, identification of cells with three different Ly phenotypes (Ly1^+^2^+^, Ly1^+^2^−^, and Ly1^−^2^+^) in the thymus (11, 12) opened new possibilities to study T cell development and intra-thymic mechanisms of selection. Years later monoclonal antibodies ousted the use of antisera. CD4, the newly discovered T cell specific molecule (13, 14) and functional counterpart of Ly2 (CD8) on Ly2^−^ T cells turned out to better define T cell subsets than Ly1(CD5), which was later shown to be also expressed at a low level on Ly2^+^ T cells and on some B cells (15). Consequently, subsets originally defined by Ly antisera as Ly1^+^2^−^ and Ly1^−^2^+^ became re-defined and re-named as CD4^+^8^−^ and CD4^−^8^+^.

Our results, which I publicly reported for the first time in summer 1974 at the workshop chaired by Martin Raff during the International Congress of Immunology in Brighton, UK, were obtained between December 1972 and August 1973. Although immediately recognized as very important (Dr. Boyse in his letter to WHO, dated August 7, 1973 wrote: “*Dr. Kisielow together with two other members of our group here has begun what may be a very important finding in the immunology of lymphocytes. Identification of different lines of T lymphocytes that may perform different function is a crucial step in our understanding of immunological responses and hence is of relevance to cancer no less than to several other fields of medicine*”) the publication in Nature (11) was delayed by non-scientific reasons.

After my return to Poland, Harvey Cantor repeated, confirmed, and extended our results by providing firm evidence for antigen independent generation of Ly1^+^2^−^ and Ly1^−^2^+^ subsets (12). In addition, he showed that Ly1^+^2^−^ lymphocytes cooperate not only with B cells but also with Ly1^−^2^+^ lymphocytes in the generation of killer activity and obtained the first suggestive evidence that antigen recognition by Ly1^+^2^−^ and Ly1^−^2^+^ may be restricted by different classes of MHC antigens (16). This was around the time of discovering the MHC restriction phenomenon (17) and some time later it was firmly established that antigen recognition by CD4^+^8^−^ and by CD4^−^8^+^ T cells is restricted by class II and class I MHC molecules respectively, and that CD4 and CD8 molecules specifically interact with restricting MHC molecules on antigen-presenting cells (18). Puzzling, for a long time unanswered questions concerned the identity of the immediate precursor cells of CD4^+^8^−^ and CD4^−^8^+^ lineages and the mechanism of their intra-thymic selection. Many researchers, including myself (19), tried to decipher the developmental potential of the obvious candidate cell, i.e., CD4^+^8^+^ (Ly1^+^2^+^) thymocyte. My desire to study this problem started my long time collaboration with Harald von Boehmer, who generously kept inviting me, and my students, to the Basel Institute for Immunology. There, we were fortunate to participate in the project aimed at studying T cell development and selection in TCR transgenic mice, which led to the identification of CD4^+^8^+^ thymocytes as a target of positive (20–22) and negative (23, 24) selection allowing us to gain insight into the mechanisms of these processes. Learning how T cell subsets, which we had identified 15 years earlier (11) are born in the thymus was one of the happiest experience in my scientific life, despite being aware that efforts to understand the immune system represent a continuous, never-ending asymptotic process where the answer to the given question is as good as the number of new questions it generates.

## Conflict of Interest Statement

The author declares that the research was conducted in the absence of any commercial or financial relationships that could be construed as a potential conflict of interest.

## Footnotes

^1^Our failure to block cytotoxicity by Ly2 antisera was probably due to the idiosyncratic properties of our method since some years later E. Nakayama and colleagues achieved blocking by measuring chromium release from labeled non- adherent target cells. (Nakayama, E., Shiku, H., Stockert, E., Oettgen H. F. and Old L. J. Cytotoxic T cells: Lyt phenotype and blocking of killing activity by Lyt antisera. (1979) *Proc. Natl. Acad. Sci*. 76: 1977–81).
